# Features of the Defect Structure of LiNbO_3_:Mg:B Crystals of Different Composition and Genesis

**DOI:** 10.3390/ma18020436

**Published:** 2025-01-18

**Authors:** Roman A. Titov, Alexandra V. Kadetova, Diana V. Manukovskaya, Maxim V. Smirnov, Olga V. Tokko, Nikolay V. Sidorov, Irina V. Biryukova, Sofja M. Masloboeva, Mikhail N. Palatnikov

**Affiliations:** 1 Tananaev Institute of Chemistry–Subdivision of the Federal Research Centre “Kola Science Centre of the Russian Academy of Sciences” (ICT KSC RAS), Apatity 184209, Murmansk Region, Russia; r.titov@ksc.ru (R.A.T.); ttyc9@mail.ru (A.V.K.); m.smirnov@ksc.ru (M.V.S.); n.sidorov@ksc.ru (N.V.S.); oksanakrav@mail.ru (I.V.B.); sofia_masloboeva@mail.ru (S.M.M.); m.palatnikov@ksc.ru (M.N.P.); 2 Solid State Physics Department, Petrozavodsk State University (PetrSU), Petrozavodsk 185910, Republic of Karelia, Russia; solvak@yandex.ru

**Keywords:** lithium niobate, doping, melt dilution method, magnesium, boron, model calculations, defects

## Abstract

We proposed and investigated a refinement of technology for obtaining Mg-doped LiNbO_3_ (LN) crystals by co-doping it with B. LN:Mg (5.0 mol%) is now the most widely used material based on bulk lithium niobate. It is suitable for light modulation and transformation. We found that non-metal boron decreases threshold concentrations of the target dopant in many ways. In addition, we earlier determined that the method of boron introduction into the LN charge strongly affects the LN:B crystal structure. So we investigated the point structural defects of two series of LN:Mg:B crystals obtained by different doping methods, in which the stage of dopant introduction was different. We investigated the features of boron cation localization in LN:Mg:B single crystals. We conducted the study using XRD (X-ray diffraction) analysis. We have confirmed that the homogeneous doping method introduces an additional defect (Mg_V_) into the structure of LN:Mg:B single crystals. Vacancies in niobium positions (V_Nb_) are formed as a compensator for the excess positive charge of point structural defects. According to model calculations, boron is localized in most cases in the tetrahedron face common with the vacant niobium octahedron from the first layer (V_Nb_^I^O_6_). The energy of the Coulomb interaction is minimal in the LN:Mg:B crystal (2.57 mol% MgO and 0.42 × 10^−4^ wt% B in the crystal); it was obtained using the solid-phase doping technology. The solid-phase doping technology is better suited for obtaining boron-containing crystals with properties characteristic of double-doped crystals (LN:Mg:B).

## 1. Introduction

Lithium niobate single crystal (LiNbO_3_, LN) is a ferroelectric nonlinear optical material that has attracted the attention of researchers for many decades due to its unique combination of properties and possible applications [1,2,3,4,5,6,7]. Recent studies have shown that LN single crystals are promising for a large number of applications. They can be used in the development of materials for holographic recording of information and coherent optical information processing systems [8,9,10,11], for generating terahertz radiation [12], for biomedicine [13], for nanoplasmonics [14,15], for creating compact crystalline accelerators that implement the generation of electron beams and soft X-ray radiation [16], for optical manipulation of micro- and nanoparticles [17,18] and water droplets [19,20,21]. Since LN is a phase of variable composition, the unique characteristics of the crystal are determined by the state of imperfection of its oxygen-octahedral structure. The Mg-doped and co-doped crystals are of the greatest interest, and materials based on LN:Mg find the widest application among all bulk LN [1,2,3,4,9,10,11]. However, practically important devices are made of Mg-doped bulk LN:Mg (5.0 mol%). This strongly suppresses optical damage and makes it suitable for laser radiation transformation as a part of periodically poled LN (PPLN) devices [10,11]. An amount of 5.0 mol% Mg in LN is considered an optimal concentration that balances useful properties and amount of defects. Meanwhile, 5.0 mol% is very close to the concentration threshold of 5.5 mol% MgO. Crystals with the dopant concentration above threshold change their properties and physical characteristics very sharply, which includes appearance of a great number of defects [2,9]. The defects prevent the use of above-threshold LN crystals in optics. Moreover, the closer the Mg concentration is to the threshold, the greater is the number of such defects, and 5.0 mol% still generates plenty of defects. Thus, it is of great relevance to find such a technology, that would provide LN:Mg crystals with the given properties but with the least possible defectivity. And that is the aim of this paper.

**Figure 1:**
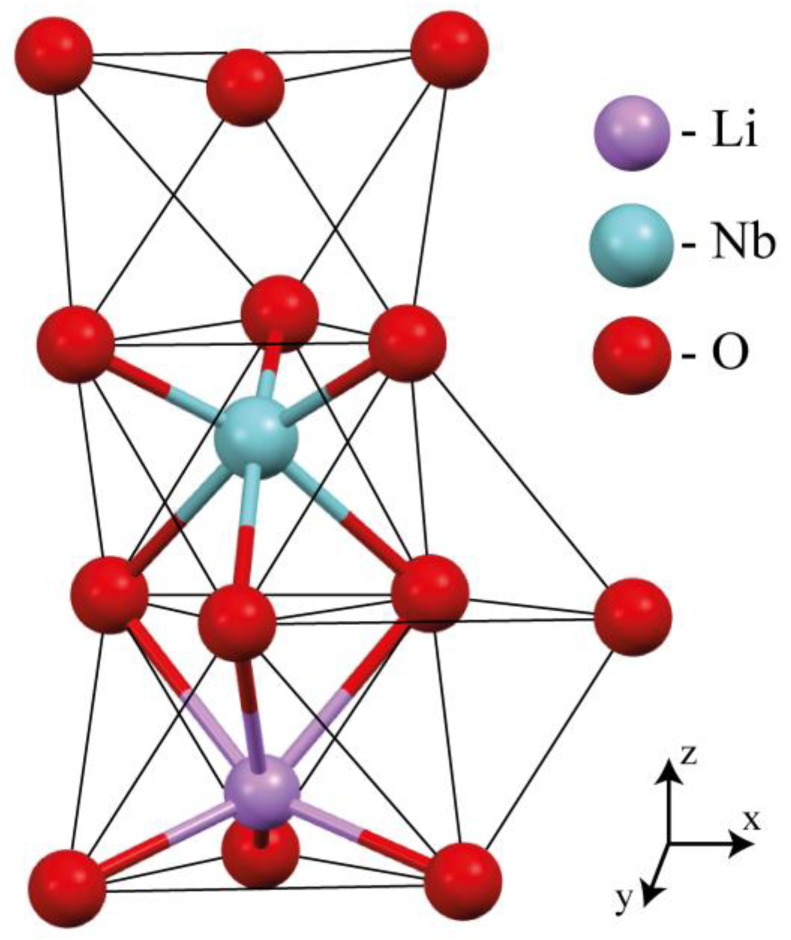
A fragment of the LiNbO_3_ crystal structure which shows the relative positions of the oxygen octahedra O_6_ (vacant and containing Li or Nb cations) and the tetrahedra O_4_.

The crystal anionic structure contains big oxygen octahedra and small-volume vacant tetrahedral voids, Figure 1. Metallic cations are usually introduced into the O_6_ octahedral voids of LN. The influence of small-volume tetrahedral voids O_4_ of the LiNbO_3_ crystal on the formation of the defect structure remains unknown. Such features of the defect structure can seriously affect its physical characteristics. And metal cations cannot be introduced into the tetrahedral voids of O_4_ due to their large ionic radius.

The phase diagram of the Li_2_O-Nb_2_O_5_ system has a number of features [22]. Obtaining LN crystals of stoichiometric composition is complicated. However, technologies have now been developed that make it possible to grow LN crystals of stoichiometric (SLN) and near-stoichiometric (NSLN) composition. One approach is to grow crystals from a melt with an excess of an alkaline component (58.6 mol% Li_2_O) [1]. The disadvantages of this method are the low growth rate and large non-uniformity of the refractive index along the crystal growth axis. The VTE (Vapor Transport Equilibrium) method involves the diffusion of gaseous lithium into the structure of an LN sample [9,23]. This method increases the stoichiometry of only thin plates; therefore, it is unsuitable for large crystals. Another approach is the HTTSSG (High-Temperature Top Seeded Solution Growth) technology. It consists of growing crystals from a charge of congruent composition (R = [Li]/[Nb] = 0.946) with the addition of alkali metal fluxes, in particular K_2_O [24,25]. The main disadvantage of the method is the high concentration of potassium (1–2 × 10^−2^ wt%) in the grown crystals. 

An alternative technological approach for obtaining NSLN is the addition of boron-containing doping components (B_2_O_3_, H_3_BO_3_) to a charge of congruent composition [26,27]. Boron-containing crystals of lithium niobate LiNbO_3_:B (hereinafter referred to as LN:B) have high structural and compositional uniformity and a low photorefractive effect. The non-metallic element boron is included in the crystal structure at the level of trace amounts (~4 × 10^−4^ mol%) and is localized in the faces of vacant tetrahedral voids [26]. The chemically active element boron structures the melt, binds excess niobium, regulates the cations of impurity metals inevitably present in the charge and prevents their transition into the structure of the growing crystal [26,27]. Such NSLN crystals should have a coercive field value as low as SLN, which is also crucial for the conversion of laser radiation in PPLN.

Currently, PPLN devices are made from LN crystals heavily doped with magnesium (≈5.0 mol% MgO) [28,29,30]. The compositional homogeneity of such crystals can decrease [31] due to the formation of a large number of structural defects [32].

Let us consider possible point defects and the models of their distribution. According to the Li-vacancy compensation model, in the crystal lattice of a congruent LN (CLN), there are ~1 mol. % Nb_Li_ point defects and ~4 mol % V_Li_ point defects [33,34]. Nb_Li_ defects disrupt the ideal alternation of structural units of the cation sublattice in LiNbO_3_ crystals; the defects cause the photorefraction effect. To reduce the concentration of such defects, optical-damage-resistant ions (ODRIs) are deliberately introduced into the LN charge. This is a group of metal cations, for example, Zn^2+^, Mg^2+^, In^3+^, etc. [2,35,36,37,38]. However, such doping can not only reduce the concentration of Nb_Li_ defects but also form other defects (Nb_V_, Me_Li_, Me_V_).

Thus, the classical ODRI dopant (Mg) reduces the photorefraction effect and the content of Nb_Li_ defects, and a non-metal (B) affects the “crystal-melt” system, the content of Nb_Li_ defects and the optical properties of such crystals. The mutual influence of these two dopants would be a fruitful approach to obtaining LN materials suitable for PPLN application. However, it should be handled with care, as an unexpected synergetic effect can arise. This means that approaches to the co-doping technology, appearing defects and their combinations should be investigated thoroughly [39,40].

The influence of boron on the structural features of LN crystals can be traced using Raman spectra. For example, Raman spectra revealed a distortion of the anion sublattice of LN:B crystals in work [41]. This was not observed in CLN and SLN crystals. A more informative method may be XRD. It provides reliable information on the coordinates of atoms and their spatial arrangement in the crystal lattice. This helps to determine the features of the localization of point defects in the crystal structure. The results of such studies, the main objects of which were LN:B crystals and some LiNbO_3_:Mg:B crystals, are given in [26,27,39]. However, XRD can be used to determine the position of atoms only heavier than boron.

Calculations of the Coulomb interaction can be both an independent method and part of a larger modeling; it is an important and indispensable tool in modern materials science. For example, the Coulomb interaction was applied and studied in various fields of materials science [42,43,44,45,46,47,48,49,50,51]. It is especially interesting that computer simulation including Coulomb interaction calculations were applied to study Mg-doped LN [52,53,54].

The work [26] determined the features of localization of boron in the structure of LN:B crystals obtained using homogeneous and direct doping technologies. The approach consisted of calculating the energy of the Coulomb interaction of point charges of fragments (clusters) of the crystal structure constructed on the basis of XRD data [27], with a boron cation. Boron was placed in different faces of vacant tetrahedral voids. Having analyzed 10 calculated clusters of different configurations, we established that in LN:B crystals, boron is localized preferentially in the faces of tetrahedra common with vacant niobium octahedra (V_Nb_) [26]. This approach can provide important information about the features of boron localization in the structure of LN:B crystals, in particular, double-doped LN:Mg:B crystals containing point structural defects. The calculations in this work were also compared with the data on monodoped crystals LN:B(1-HG) and LN:B(2-SP) obtained in [26]. LN:B(1-HG) crystal was obtained by homogeneous doping, and LN:B(2-SP) by solid phase.

Thus, the aim of this work is to study the structural features of LN:Mg:B crystals of different genesis. We grew crystals from a charge obtained using homogeneous (HG) and solid-phase (SP) doping technologies in this work. The crystals were grown by stepwise dilution of the initial melt. The study was carried out by the Rietveld refinement of XRD patterns and using model calculations. Calculations revealed the features of localization of B^3+^ ions in the faces of vacant tetrahedral voids of such crystals, taking into account the detected point defects. An important task of the study is also the justification of the choice of the optimal doping technology for obtaining LN:Mg:B crystals with the least amount of structural defects.

## 2. Materials and Methods

Single crystals of LN:Mg:B(1-4-HG) and LN:Mg:B(5-8-SP) with a diameter of 35–38 mm and a cylindrical part length of 35–40 mm were grown in the (001) direction in an air atmosphere from platinum crucibles with a diameter of 85 mm by the Czochralski method on a growth setup Kristall-2 (Voroshilovgradsky zavod electronnogo mashinostroeniya, Voroshilovgrad, USSR). The setup is equipped with a thyristor generator and an automated control system. The thermal conditions and technological modes of growing single crystals are given in [39]. Numbers in designations of crystals LN:Mg:B(1-4-HG) and LN:Mg:B(5-8-SP) mean number of a crystal. Further explanations and details are given below.

Two types of charges were used to grow LN:Mg:B(1-8) single crystals. We used a homogeneously doped charge for growing LN:Mg:B(1-4-HG) crystals [55]. Boron and magnesium were introduced at the stage of niobium hydroxide precipitation from high-purity niobium-containing fluoride solutions. After that, we carried out high-temperature synthesis granulation of the Nb_2_O_5_:B:Mg-Li_2_CO_3_ mixture.

We used a solid-phase doped charge for growing LN:Mg:B(5-8-SP) crystals [56]. Boron and magnesium were introduced at the stage of preparation of the Li_2_CO_3_-Nb_2_O_5_-H_3_BO_3_-MgO mixture. This mixture is made in order to carry out the synthesis granulation of the charge. Boron was added in the form of boric acid (H_3_BO_3_), magnesium was added in the form of MgO (high purity, concentration of foreign impurities at the level of <5 × 10^−4^ wt%) [39]. In both cases, the following substances were used for the synthesis of the charge: niobium pentoxide Nb_2_O_5_ grade A produced using Technical Specifications 1763-025-00545484-2000 at Solikamsk magnesium works (Solikamsk, Russian Federation); high-purity lithium carbonate Li_2_CO_3_ with a concentration of impurities at a level of <3 × 10^−4^ wt% [39]. To synthesize the HG charge, HF (99.99, Vekton Ltd., Saint Petersburg, Russia) and NH_4_OH (25% solution, Komponent-reaktiv Ltd., Moscow, Russia) were used.

When calculating the required amount of Li_2_CO_3_ to obtain a LiNbO_3_ charge of congruent composition (R = 0.946), attention was paid to the magnesium content in the mixture. The concentration of boron can be compared with the concentration of trace amounts of impurities [26,41]. Therefore, it was not taken into account when determining the amount of Li_2_CO_3_. Synthesis granulation of mixtures (Nb_2_O_5_:B:Mg-Li_2_CO_3_ and Li_2_CO_3_-Nb_2_O_5_-H_3_BO_3_-MgO) was carried out at a temperature of ~1235–1245 °C for 5 h. The heating rate of the mixtures was ~200 °C/h [39].

Two series of crystals LN:Mg:B(1-4-HG) and LN:Mg:B(5-8-SP) were grown from the HG- and SP-doped charges by stepwise dilution of the initial melt. After growing each LN:Mg:B single crystal, the nominally pure lithium niobate charge of congruent composition was added to the melt remaining in the crucible [39]. Table 1 shows the content of doping elements in the grown crystals of LN:Mg:B(1-4-HG) and LN:Mg:B(5-8-SP). The content of dopants in the melt is given in [39,40]. The crystals were grown in the following order: LN:Mg:B(4-HG) → LN:Mg:B(3-HG) → LN:Mg:B(2-HG) → LN:Mg:B(1-HG); LN:Mg:B(8-SP) → LN:Mg:B(7-SP) → LN:Mg:B(6-SP) → LN:Mg:B(5-SP).

Table 1 shows that in LN:Mg:B(3-HG) and LN:Mg:B(8-SP) crystals, the magnesium concentrations are very close to each other.

The concentration of magnesium in the charge and crystals was determined by AES (ICPE-9000, Shimadzu, Japan, Kyoto, 2011) with an accuracy of 4 × 10^−3^%. The boron content was determined by inductively coupled plasma mass spectrometry (ELAN 9000 DRC-e, PerkinElmer, Hopkinton, MA, USA) with an accuracy of 1 × 10^−6^%.

To carry out Rietveld refinement, XDR patterns of the studied crystals were recorded on a DRON-6 diffractometer (NPP Burevestnik, Saint Petersburg, Russian Federation) in monochromatic CuKα radiation (λ = 1.54178 Å) in the range of scattering angles 2θ from 5 to 145°. The Rietveld method was used to determine the unit cell parameters, the position of the doping element (Mg) in the structure, the type of intrinsic defects and their concentration. The MRIA and FULL PROF programs were used.

During the Rietveld refinement of powder XRD patterns of the LN:Mg:B(1-4-HG) and LN:Mg:B(5-8-SP) samples studied in the work, all possible models of the arrangement of intrinsic [57,58,59] and doping defects in the structure of the lithium niobate crystal were refined. Magnesium doping atoms were located in the positions of lithium (Mg_Li_), niobium (Mg_Nb_) and the empty octahedron (Mg_v_). The position of boron in the structure was not specified due to its low concentration (~10^−3^ mol%) in the studied samples. A total of 30 probabilistic models of defect structure were refined for each sample. The criteria for selecting the final model for describing the defect structure were the minimum values of the agreement factors (*R_w_*, *R_wp_*), the stability of the refined parameters during Rietveld refinement, and the values of electroneutrality. The latter is calculated using the formula A + 5B + 2C = 6, where A is the number of lithium atoms, B is the number of niobium atoms in the main position, and in the defect node, C is the number of magnesium atoms.

The calculation of the total energy of the Coulomb interaction of point charges (*E_C_*, eV) of the fragment of the structure of LN:Mg:B(1-4-HG) and LN:Mg:B(5-8-SP) with the considered cation B^3+^ in the *sp*^2^-hybrid state was carried out using the Coulomb potential [26]:

(1) $$E_{C}=k\cdot \sum_{i=1}^{n}\frac{q_{i}\cdot q_{B}}{r_{iB}}$$

where *q_i_* and *q_B_* are the values of the charge of the interacting particles in fractions of an electron; *r_iB_* is the distance between the centers of the interacting charges [Å]; and *k* is a constant (eV⋅Å), expressed by the formula [26]:

k = e^2^/(4 · π · ε_0_ ·10^−10^) = 14.41971 (2)

where *e* is the electron charge, and ε_0_ is the dielectric constant.

When calculating the energy of Coulomb interaction, we used data obtained by X-ray structural analysis. The number of significant figures after the decimal point is 3. This determines the accuracy of the calculations.

The system (cluster) under consideration includes six oxygen octahedra in two layers: lower (I) and upper (II). For example, the notation for the placement of intrinsic cations in a cluster without defects and with ideal order would be as follows: Li^I^O_6_, Nb^I^O_6_, V^I^O_6_, Li^II^O_6_, Nb^II^O_6_ and V^II^O_6_. Filling the constructed O_6_ octahedra with Li and Nb cations corresponds to filling the real LN:Mg:B(1-4-HG) and LN:Mg:B(5-8-SP) crystals with these cations. The composition of such a cluster takes into account two tetrahedral voids formed by the selected oxygen octahedra. It should be noted that the system under consideration is not electrically neutral. In this work, we study the change in the energy of the Coulomb interaction of the B^3+^ cation with the surrounding fragment of the real crystal structure of LN:Mg:B(1-4-HG) and LN:Mg:B(5-8-SP) crystals. We included in the consideration the defects Nb_Li_, Nb_V_, Mg_Li_, Mg_V_ and V_Nb_. The position of B^3+^ was taken to be equidistant from the oxygen atoms in each face of the vacant tetrahedral voids. The cluster framework is formed by 20 oxygen anions O^2–^. The number and localization of the main metal cations (Li^+^ and Nb^5+^) in the clusters differ and depend on the coordinates of the atoms, the type and number of defects introduced into the cluster. Structural defects Nb_Li_, Nb_V_, Mg_Li_ and Mg_V_ were detected in LN:Mg:B(1-4-HG) and LN:Mg:B(5-8-SP) crystals during XRD patterns analysis. These defects determined the choice of models for describing the localization of intrinsic point defects in the studied crystals: the niobium vacancy model (M2 [35,59]) and the model of filling empty octahedra (M3 [1,59]).

In this work, we considered the formation of the following defects of different types: Nb_Li_^4•^ (niobium cation localized in the lithium octahedron), Nb_V_^5•^ (niobium cation localized in the vacant octahedron), Mg_Li_^•^ (magnesium cation localized in the lithium octahedron), Mg_V_^2•^ (magnesium cation localized in the vacant octahedron), V_Nb_^5′^ (vacancy in the niobium octahedron). The vacancy in the niobium octahedron is formed within the framework of charge compensation of the defects Nb_Li_^5+^, Nb_V_^5+^, Mg_Li_^2+^ and Mg_V_^2+^. Since the model calculations were performed using a limited fragment of the crystal structure (6 octahedra), we took into account the point charge of the defects introduced into the cluster, i.e., Nb_Li_^5+^, Nb_V_^5+^, Mg_Li_^2+^ and Mg_V_^2+^. The point charge may differ from the defect charge relative to the lattice.

The methodology for evaluating the influence of doping technology and the set of defective clusters on the features of the localization of the boron cation in the faces of vacant tetrahedral voids are given in [26].

For convenient description of the considered clusters, we introduce the following designations: «A.B.C», Table 2. In this designation, «A» is the crystal number (according to Table 1), «B» is the type of the considered cluster (0—without defects, 1—contains one Nb_Li_ defect, 2—contains one Nb_V_ defect, 3—contains one Mg_Li_ defect, 4—contains one Mg_V_ defect), «C» is the position of the considered defect (Nb_Li_, Nb_V_, Mg_Li_ or Mg_V_) in the cluster (0—the defect is absent, 1—the defect is located in the 1st layer of the cluster, 2—in the 2nd layer of the cluster). For example, the entry «3.0.0» means that the defect-free cluster of crystal 3 is considered; the entry «4.2.1» means that the cluster of crystal 4 is considered, in which the Nb_V_ defect is located in the 1st layer.

As a result, 64 clusters were constructed and calculated based on XRD analysis data:

- 8 defect-free clusters (1.0.0–8.0.0);
- 16 clusters containing an Nb_Li_ defect (1.1.1–8.1.1, 1.1.2–8.1.2);
- 16 clusters containing an Nb_V_ defect (1.2.1–8.2.1, 1.2.2–8.2.2);
- 16 clusters containing an Mg_Li_ defect (1.3.1–8.3.1, 1.3.2–8.3.2);
- 8 clusters containing an Mg_V_ defect (1.4.1–4.4.2).

Several possible cationic compositions of model clusters of LN:Mg:B(1-4-HG) and LN:Mg:B(5-8-SP) crystals are considered in this work. The periods of the unit cells (Table 3, Figure 1, work [40]) and the type and coordinates of the defects under consideration (Nb_Li_, Nb_V_, Mg_Li_ and Mg_V_: Table 3 and work [39]) were taken into account when forming the clusters.

## 3. Results and Discussion

### 3.1. XRD Studies

Table 3 shows the atomic coordinates (x/*a*, y/*b*, z/*c*), site occupancies (*G*), and *R*-factors obtained during Rietveld refinement. Table 3 shows the data for the samples LN:Mg:B(2, 3-HG) and LN:Mg:B(5, 6-SP); for the other samples, the data are published in [39].

Analysis of the obtained models of the location of doping defects in the studied crystals showed that the mechanism of magnesium incorporation into the structure depends on the doping technology of the samples. Magnesium occupies only lithium positions in samples grown from the SP charge, and most of the magnesium atoms are additionally located in the empty octahedron in samples grown from the HG charge (Table 3, [39]). And the doping atoms are not located in the regular position of lithium; they are shifted either to the center of the octahedron or to the oxygen plane.

All samples contain intrinsic defects: niobium in the lithium site (Nb_Li_), and niobium in the empty oxygen octahedron (Nb_v_). Charge compensation, when defects (Nb_Li_, Nb_v_, Mg_Li_, Mg_V_) arise, occurs due to the formation of niobium vacancies (V_Nb_). The number of lithium vacancies is small or equal to zero.

The concentration of Me_V_ defects in samples grown from the HG charge is higher than in samples grown from the SP charge (Table 3, [39]). The presence of defects of this type disorders the cation sublattice along the polar axis and increases the defectiveness of the structure.

**Figure 2:**
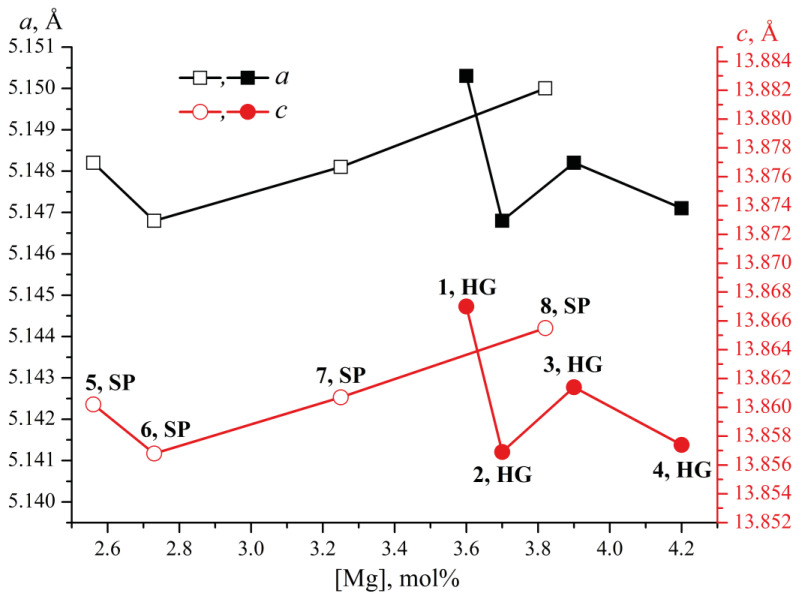
Dependence of the unit cell periods (*a* and *c*, Å) on the magnesium concentration in LN:Mg:B(1-4-HG) (bulk dots) and LN:Mg:B(5-8-SP) (empty dots) crystals. Data for LN:Mg:B(1,4-HG) and LN:Mg:B(5-8-SP) crystals are taken from [40]).

The periods and volume of the unit cell for all the studied double-doped crystals exceed the corresponding values for the NSLN (HTTSSG) crystal (*a* = 5.1428 Å, *c* = 13.8443 Å, *V* = 317.10 Å^3^ [39]). In crystals grown from HG charge, a non-monotonic dependence of the values of the unit cell periods on the dopant concentration in the crystal is observed, Figure 2. In crystals grown from the SP charge, when the magnesium concentration increases, the unit cell volume first decreases (LN:Mg:B(6-SP) crystal), and then the volume increases (LN:Mg:B(7 and 8-SP) crystals), Figure 2. The highest period value is achieved in the LN:Mg:B(8-SP) crystal at the maximum magnesium concentration in this series. The opposite trend is observed in crystals grown from the HG charge: the highest period value is recorded in the LN:Mg:B(1-HG) crystal which contains the minimum magnesium concentration in this series.

### 3.2. Coulomb Energy Calculation Studies

**Figure 3:**
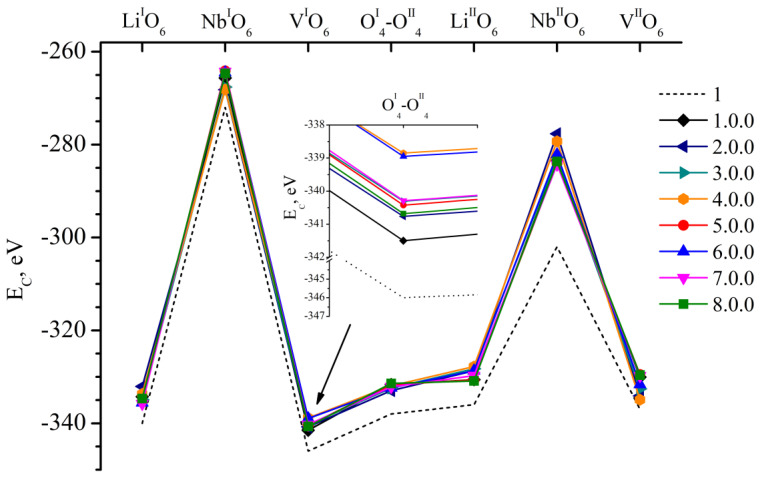
Dependence of *E_C_* on the position of the B^3+^ cation in the faces of the tetrahedrons of clusters 1.0.0–8.0.0, and in control Cluster 1 [26]. The positions of boron in the clusters under consideration are indicated on the abscissa axis. A slanted arrow indicates which part of the graph is enlarged at an inset.

Figure 3 shows the *E_C_* value of clusters 1.0.0–8.0.0. These clusters correspond to fragments of LN:Mg:B(1-4-HG) and LN:Mg:B(5-8-SP) crystals. They contain a boron cation in one of the seven faces of vacant tetrahedral voids considered in this work. The ideal alternation of the main metals (Li, Nb) and vacancies (V) along the polar axis of the crystal is considered for lithium niobate in these clusters. The alternation excludes the presence of point structural defects. Figure 3 additionally demonstrates the dependence of *E_C_* of the comparison Cluster 1. It is constructed on the basis of XRD structural data [1] for the CLN crystal. We obtained the data in an earlier study [26]. The *E_C_* for all possible positions of the boron cation in the faces of vacant tetrahedral voids of the clusters 1.0.0–8.0.0 is greater than for the control cluster, Figure 3. This can be explained by a more multicomponent “crystal-melt” system during the production of LN:Mg:B(1-4-HG) and LN:Mg:B(5-8-SP) crystals, in contrast to the melt of a congruent composition which is simpler in composition. To synthesize the doped charge, which was used to grow LN:Mg:B(1-4-HG) and LN:Mg:B(5-8-SP) crystals, in addition to the main components Li_2_CO_3_ and Nb_2_O_5_, MgO and H_3_BO_3_ were added to the reaction mixture. Such additives influence the type and concentration of electrochemical complexes in the melt. They determine the features of both the primary and secondary structure of the grown crystals.

The *E_C_* values of boron cations for clusters 1.0.0–8.0.0 have fairly close values (Δ_max_ *E*_C_ = 6 eV) in all positions considered, Figure 3. No significant differences in *E_C_* that could be explained by the technology of obtaining crystals and different concentrations of doping elements in them could be detected in the case of Clusters 1.0.0–8.0.0. The minor difference in *E_C_* found is due to the individual characteristics of each crystal (different periods of the unit cell, different distances between cations and anions of the clusters). More significant differences in *E_C_* will be shown further when considering point structural defects in clusters.

**Figure 4:**
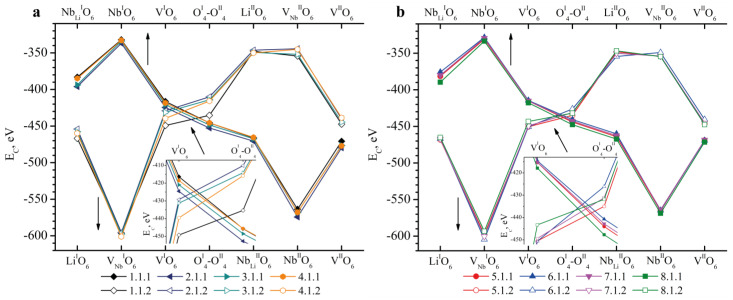
Dependence of *E_C_* on the position of the B^3+^ cation in the tetrahedron face in clusters containing the Nb_Li_ defect: (**a**)—1.1.1–4.1.2; (**b**)—5.1.1–8.1.2. The abscissa axis in the upper part of the figures shows the positions of boron in Clusters 1.1.1–8.1.1 (bulk dots), and the lower part of the figures shows the positions of boron in Clusters 1.1.2–8.1.2 (empty dots). A slanted arrow indicates which part of the graph is enlarged at an inset. Straight arrows indicate which abscissa axis (upper or lower) should be taken into account when analyzing the graph.

Figure 4a,b shows the results of *E_C_* calculations in Clusters 1.1.1–8.1.1 (abscissa axis in the upper part of the graph) and 1.1.2–8.1.2 (abscissa axis in the lower part of the graph). Recall that in Clusters 1.1.1–8.1.1, the Nb_Li_ defect is located in layer I, and V_Nb_ is located in layer II; in Clusters 1.1.2–8.1.2, the Nb_Li_ defect is located in layer II, and V_Nb_ is located in layer I. Common to all the clusters considered is the fact that the most energetically favorable position of boron is its localization on the face of the tetrahedron adjacent to the vacant niobium octahedron (V_Nb_^I^O_6_), Figure 4. The *E_C_* of Clusters 1.1.2–8.1.2 for the V_Nb_^I^O_6_ position is in the range from −593 to −604 eV, respectively. The E_C_ of Clusters 1.1.1–8.1.1 for the V_Nb_^II^O_6_ position is in the range from −564 to −574 eV, respectively, Figure 4.

The *E_C_* of most of the possible boron positions calculated for HG crystal clusters has a large difference in values when compared with the *E_C_* calculated for SP crystal clusters (Figure 4).

For example, the *E_C_* for the V^I^O_6_ position in Clusters 1.1.2–4.1.2 of HG crystals varies from −429 to −449 eV, respectively (Figure 4). And the *E_C_* for SP crystals (Clusters 5.1.2–8.1.2) for the V^I^O_6_ position varies in a wider range from −443 to −451 eV, respectively. The *E_C_* for the O^I^_4_-O^II^_4_ position of HG crystals also varies greatly, Figure 4a: 1.1.2 (−435 eV), 2.1.2 (−410 eV), 3.1.2 (−414 eV), 4.1.2 (−416 eV). This is not observed for the O^I^_4_-O^II^_4_ position of SP crystals, Figure 4b: *E_C_* for Clusters 5.1.2–8.1.2 varies within the range from −426 to −435 eV. Such differences in *E_C_* for similar positions of the boron cation in clusters of the same configuration, but for crystals of different genesis, can be due to the following reasons.

Firstly, the homogeneous doping technology involves the introduction of a doping element at the stage of Nb_2_O_5_ precursor preparation. On the one hand, this increases the distribution coefficient of the dopant [39], and on the other hand, it can lead to a more complex composition of the ionic complexes. They participate in the crystallization process.

Secondly, the LN:Mg:B(1-4-HG) crystal series contains a higher concentration of magnesium cations than the LN:Mg:B(5-8-SP) crystal series (3.6–4.2 versus 2.57–3.82 mol%, respectively), Table 1.

Thirdly, the *E_C_* calculation method takes into account the relative positions of the oxygen framework, main cations, and point defects (as well as their charge component). In this case, the coordinates of both the main cations and defects may differ, see Table 3 and work in [39]. And the defects are determined by the technology of production, the type and concentration of dopants and the technological parameters of crystal growth. Such differences also appear when calculating *E_C_*.

**Figure 5:**
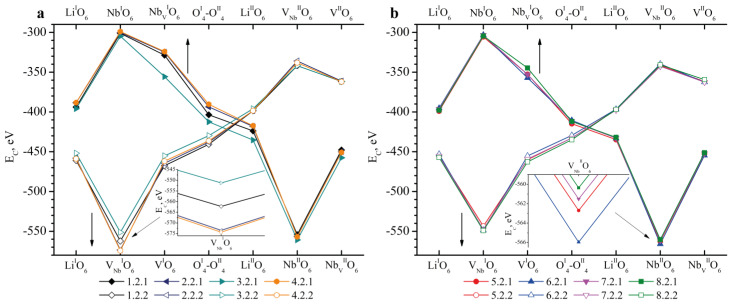
Dependence of *E_C_* on the position of the B^3+^ cation in the tetrahedron faces in clusters containing the Nb_V_ defect: (**a**)—1.2.1–4.2.2.; (**b**)—5.2.1–8.2.2. The positions of boron are indicated on the abscissa axis in the upper part of the figures in clusters 1.2.1–8.2.1 (bulk dots), in the lower part of the figures—in clusters 1.2.2–8.2.2 (empty dots). A slanted arrow indicates which part of the graph is enlarged at an inset. Straight arrows indicate which abscissa axis (upper or lower) should be taken into account when analyzing the graph.

Nb_V_ is the next defect. It was found in LN:Mg:B(1-4-HG) and LN:Mg:B(5-8-SP) crystals (Table 3) and work [39]. The results of model calculations taking into account the presence of this defect in Clusters 1.2.1–8.2.2 are shown in Figure 5a,b. As in the case of Clusters 1.1.1–8.1.2, the localization of boron is most likely in the face of the tetrahedron which is common with the vacant niobium octahedron, Figure 5. The *E_C_* for the V_Nb_^II^O_6_ position in Clusters 1.2.1–4.2.1 and 5.2.1–8.2.1 varies within the range of −554 to −561 eV and −562 to −566 eV, respectively. In turn, the *E_C_* for the V_Nb_^I^O_6_ position in Clusters 1.2.2–4.2.2 and 5.2.2–8.2.2 varies within the range of −551 to −574 eV and −544 to −549 eV, respectively. In the case of the formation of the Nb_V_ defect, the localization of the boron cation will be energetically favorable in the tetrahedron faces adjacent to V_Nb_ from both the first and second layers. In this case, the absolute minimum of the *E_C_* values corresponds to the position of V_Nb_^I^O_6_ for Clusters 2.2.2 and 4.2.2 (−573 and −574 eV, respectively).

The behavior of the dependence of *E_C_* on the localization of the boron cation in seven different positions of Clusters 1.2.1–8.2.1 is similar for different crystals. Significant differences are observed for the Nb_V_^I^O_6_, O^I^_4_–O^II^_4_, and Li^II^O_6_ positions of Cluster 3.2.1 compared to Clusters 1.2.1, 2.2.1, 4.2.1, and 5.2.1–8.2.1, Figure 5. The decrease in *E_C_* for the indicated boron positions in Cluster 3.2.1 is due to the low location of the niobium cation in the Nb_V_^I^O_6_ octahedron of the LN:Mg:B(3-HG) crystal, Table 3. As a result, the distance between the Nb_V_^I^O_6_ defect from the first layer and the boron cations in the Nb_V_^I^O_6_, O^I^_4_-O^II^_4_ and Li^II^O_6_ positions increases for this crystal. This leads to a decrease in *E_C_* for these boron positions when Cluster 3.2.1 is considered.

**Figure 6:**
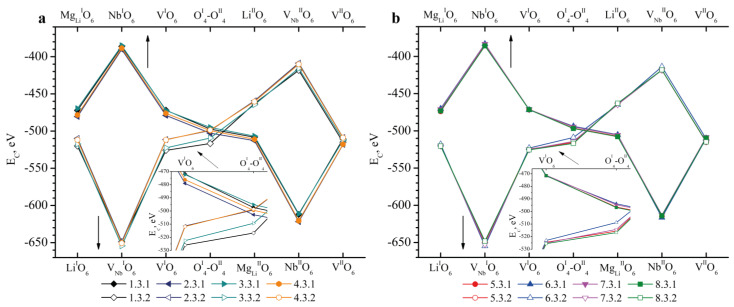
Dependence of *E_C_* on the position of the B^3+^ cation in the tetrahedron faces in clusters containing the Mg_Li_ defect: (**a**)—1.3.1–4.3.2; (**b**)—5.3.1–8.3.2. The positions of boron are indicated on the abscissa axis in the upper part of the figures in Clusters 1.3.1–8.3.1 (bulk dots), in the lower part of the figures—in Clusters 1.3.2–8.3.2 (empty dots). A slanted arrow indicates which part of the graph is enlarged at an inset. Straight arrows indicate which abscissa axis (upper or lower) should be taken into account when analyzing the graph.

The Mg_Li_ point defect was detected in all the crystals under study. As in the case of the formation of Nb_Li_ (Figure 4) and Nb_V_ (Figure 5) defects, the Mg_Li_ defect in Clusters 1.3.1–8.3.2 has a minimum *E_C_* value in cases where boron is localized near V_Nb_, Figure 6. The absolute minimum *E_C_* is characteristic of the V_Nb_^I^O_6_ position when boron is introduced into the magnesium-substituted lithium octahedron of the second layer (Mg_Li_^II^O_6_), Figure 6.

The *E_C_* remains almost unchanged when considering Clusters 1.3.1–8.3.2 with the exception of Clusters 1.3.2–4.3.2 (V^I^O_6_ and O^I^_4_–O^II^_4_ positions), Figure 6a. The Mg_Li_ defect in LN:Mg:B(2, 4-HG) crystals is localized lower than in LN:Mg:B(1, 3-HG) crystals (Table 3 and work [39]). This explains such different values of *E_C_* for the V^I^O_6_ and O^I^_4_-O^II^_4_ positions of Clusters 1.3.2–8.3.2, Figure 6a.

**Figure 7:**
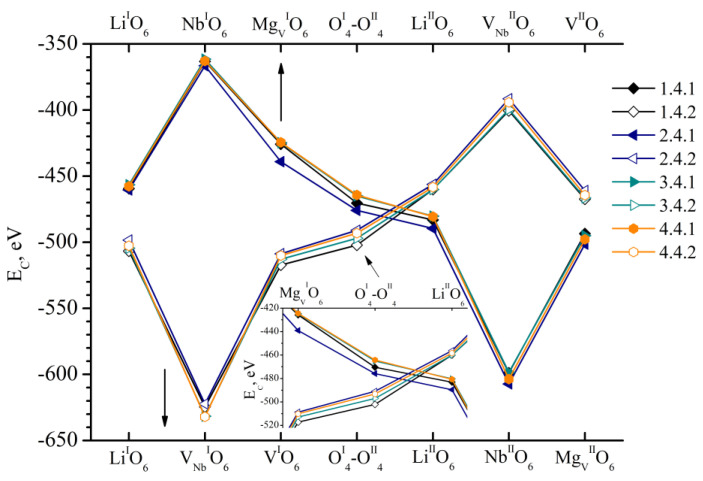
Dependence of *E_C_* on the position of the B^3+^ cation in the tetrahedron faces in Clusters 1.4.1–4.4.2 containing the Mg_V_ defect. The abscissa axis in the upper part of the figure shows the positions of boron in the clusters. A slanted arrow indicates which part of the graph is enlarged at an inset. Straight arrows indicate which abscissa axis (upper or lower) should be taken into account when analyzing the graph.

For Cluster 2.4.1, the *E_C_* in the positions of Mg_V_^I^O_6_, O^I^_4_-O^II^_4_ and Li^II^O_6_ is 7–15 eV less than the *E_C_* for the corresponding positions of boron in Clusters 1.4.1, 3.4.1 and 4.4.1, Figure 7. These differences, as in the previous cases, are explained by the peculiarity of the localization of the defect in question in the oxygen octahedron. The Mg_V_ defect in the oxygen octahedron of the LN:Mg:B(2-HG) crystal is localized lower than in the LN:Mg:B(1, 3, 4-HG) crystals (Table 3 and work [39]).

Thus, at this stage, we can conclude the following:

1. We considered Clusters 1.1.1–8.3.2 and 1.4.1–4.4.2. They contain Nb_Li_, Nb_V_, Mg_Li_ and Mg_V_ defects compensated by V_Nb_. In them, the most energetically favorable position for the boron cation and the system as a whole will be its localization in the oxygen plane adjacent to the vacant niobium octahedron. The exception is Clusters 1.2.1–8.2.2. They contain the Nb_V_ defect. For these clusters, the presence of a boron cation in the faces of vacant tetrahedral voids common with the vacant niobium octahedron from the second layer (V_Nb_^II^O_6_) will be more energetically favorable. The exception is Clusters 2.2.2 and 4.2.2, for which the minimum *E_C_* is still characteristic of the V_Nb_^I^O_6_ position, Figure 5.
2. The mutual arrangement of the structural units of the clusters (oxygen framework, main cations and structural defects) of real magnesium–boron co-doped LN crystals depends on several factors and affects the localization of boron cations in the structure. The factors are the doping technology, the type and concentration of dopants.

As mentioned above, there are several vacancy models for describing the defect structure of the LN crystal. The results of XRD analysis for boron-containing LN crystals were obtained: for LN:B(1-HG) and LN:B(2-SP) in [26]; LN:Mg:B(1, 4-HG) and LN:Mg:B(7, 8-SP) in [39]; LN:Mg:B(1, 4-HG) and LN:Mg:B(5-8-SP) in [40]; LN:Mg:B(2, 3-HG) in the present work. The results show that in the studied series of crystals, positively charged defect centers are compensated by vacancies in the niobium position. We believe that this is not a coincidence. Boron as an active complexing agent performs several actions: it binds excess niobium cations in a melt of congruent composition, complexes regulated cations of impurity metals, and structures the melt [27]. From a melt of congruent composition (with the addition of magnesium and boron or only boron), initially excess in niobium, a crystal grows which is close in stoichiometry to 1. The stoichiometry values of co-doped crystals LN:Mg:B(1-4-HG) and LN:Mg:B(4-8-SP) are 0.997, 0.971, 1.014, 0.985, 0.982, 0.973, 0.965 and 0.996, respectively, (Table 3 and work [39]). The stoichiometry values of single-doped crystals LN:B(1-HG) and LN:B(2-SP) are 0.985 and 1.033, respectively [26]. Consequently, part of the excess niobium is incorporated into lithium and vacant octahedra. To a greater extent, this mechanism is realized in LN:Mg:B(1-4-HG) and LN:Mg:B(5-8-SP) crystals (Table 3 and work [39]); to a lesser extent, it is realized in LN:B(1-HG) and LN:B(2-SP) crystals [26]). In the case of LN:Mg:B(1-4 HG) and LN:Mg:B(5-8-SP) crystals, magnesium cations additionally act as a non-photorefractive additive (ODRI) and are incorporated into lithium octahedra. Magnesium is incorporated into vacant octahedra only in LN:Mg:B(1-4-HG) crystals. As a result, the proportion of occupied niobium octahedra decreases, and V_Nb_ is formed. Thus, when boron-containing dopants appear in the reaction mixture, vacancies in the niobium position (V_Nb_^5−^) begin to compensate for the excess positive charge of point structural defects, instead of lithium vacancies (V_Li_^-^). That is, the presence of boron in the reaction mixture determines the type of vacancy model. It is worth noting that niobium vacancies are also observed in crystals doped with Er and Tb [60,61].

The lithium vacancy model cannot explain the NSLN composition of LN:Mg:B and LN:B crystals. To compensate for the positive defects, many more lithium vacancies are required than niobium vacancies. With such a number of lithium vacancies, the proportion of occupied lithium octahedra will decrease sharply which will lead to a decrease in the stoichiometry of such crystals.

**Figure 8:**
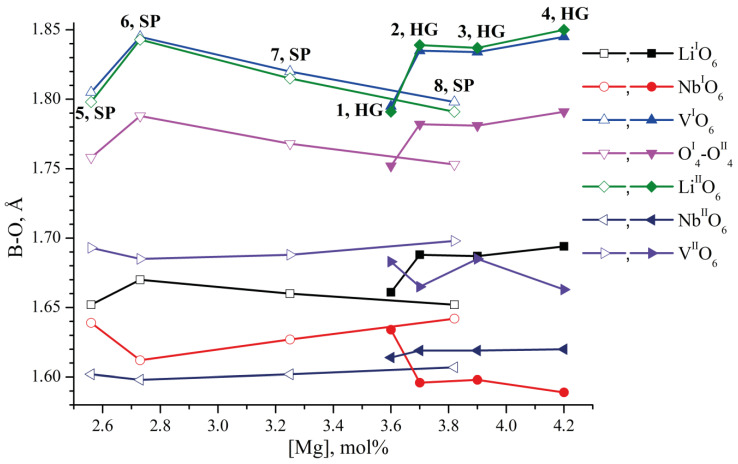
Dependence of the change in the lengths of B-O bonds (Å) in the faces of the tetrahedra of the considered clusters on the concentration of magnesium (mol%) in LN:Mg:B(1-4 HG) and LN:Mg:B(5-8-SP) crystals.

In favor of the stated hypothesis, one can consider Figure 8. It shows the dependence of the change in the lengths of B-O bonds in the faces of vacant tetrahedral voids of the considered clusters on the concentration of magnesium in LN:Mg:B(1-4 HG) and LN:Mg:B(5-8-SP) crystals. It is well known that niobium octahedra are smaller than lithium and vacant ones because five covalent bonds and one ionic bond are formed between niobium and oxygens in NbO_6_ octahedra [1]. Boron has a significantly smaller ionic radius (for trivalent B^3+^—0.15 Å [62]) than oxygen (1.26 Å [62]). Therefore, it is difficult for boron to stay in the large-area faces of the tetrahedron. These are the faces common with the octahedra Li^I^O_6_, Li^II^O_6_, V^I^O_6_, V^II^O_6_ and the face O^I^_4_-O^II^_4_. The area of the faces common with Nb^I^O_6_ and Nb^II^O_6_ is much smaller (Figure 8). Boron stays in them better.

Thus, the localization of boron near vacant niobium octahedra can be explained from several sides at once. This localization is explained by the change in the stoichiometry of the doped charge of congruent composition, the difference in the charges of B^3+^ and V^I/II^_Nb_^5−^ and the steric factor.

We have considered particular cases of real crystals in our paper. These cases include a specific defect. It was detected by the X-ray structural analysis method. The defect is compensated by V_Nb_. However, the proportion of such “defective” clusters in the structure of a real crystal is very small. Therefore, in order to evaluate the influence of the set and number of the considered defect-containing clusters on the total *E_C_* in each specific position of the boron cation (seven positions), it is necessary to take into account the population factors *G* of the positions of the intrinsic metal cations (Li, Nb), defects (Nb_Li_, Nb_V_, Mg_Li_, Mg_V_) and the proportion of vacant (V_V_) and vacant niobium octahedra (V_Nb_) (Table 3 and work [39]). This differs from the case when the ideal filling of the cluster with basic cations and vacancies is considered without taking into account defects (Figure 3). Therefore, we approach the next stage of adaptation of real X-ray structural data.

The formula for the calculation of the real contribution of energy of each boron position (*E_GC_*) to the whole crystal 1 is as follows (Cluster 1.0.0 is given as an example):

(3) $$E_{GC(1.0.0/{Li}^{I}O_{6})}=E_{C(1.0.0/{Li}^{I}O_{6})}\cdot \;K_{1.0.0}$$

where $E_{GC(1.0.0/{Li}^{I}O_{6})}$ is the Coulomb interaction energy of a boron cation located in the Li^I^O_6_ face of a tetrahedron of Cluster 1.0.0, $E_{C(1.0.0/{Li}^{I}O_{6})}$ is a corresponding value from Figure 3, and *K_1.0.0_* is a coefficient taking into account the site population factors of the main metal cations and defects in the structure of the considered Cluster 1.0.0 (see [39]).

The coefficient is calculated using the formula:

*K_1.0.0_* = *G*(Li_Li_) · *G*(Nb_Nb_) · *G*(V_V_) (4)

where *G*(Li_Li_) is the population factor of Li atoms in lithium sites, *G*(Nb_Nb_) is the population factor of Nb atoms in niobium sites, and *G*(V_V_) is the amount of strictly vacant octahedra in vacant sites calculated using the Formula (5):

*G_1–4_*(V_V_) = 1 − *G*(Nb_V_) − *G*(Mg_V_) (5)

where *G*(Nb_V_) is the population factor of Nb atoms occupying vacant octahedra, and *G*(Mg_V_) is the population factor of Mg atoms occupying vacant octahedra (only for crystals 1–4).

Strictly speaking, we cannot discuss vacancy ‘population’, since these are octahedra free of any cations; they are not populated. However, LN structure vacancies are crucial; we must take their amount into consideration.

Calculations of *EGC(i)* and *Ki* for all seven possible boron positions (in Li^I^O_6_, Nb^I^O_6_, V^I^O_6_, O^I^ _4_-O^II^_4_, Li^II^O_6_, Nb^II^O_6_, and V^II^O_6_ faces of tetrahedra), made for Cluster 1.0.0 according to Formulas (3)–(5), will be similar for Clusters 2.0.0–4.0.0. For Clusters 5.0.0–8.0.0 in (5) *G*(V_V_) calculation considers only *G*(Nb_V_):

*G_5–8_*(V_V_) = 1 − *G*(Nb_V_) (6)

The calculations for Clusters 1.1.1–8.1.1, 1.1.2-8.1.2 were similar, but they included data for the corresponding defect (Nb_Li_) and compensated for the niobium vacancy (V_Nb_). The formula for these clusters is given as an example, and was calculated correspondingly (Cluster 1.1.1 is given as an example):

(7) $$E_{GC(1.1.1/{{Nb}_{Li}^{I}O}_{6})}=E_{C(1.1.1/{Nb}_{Li}^{I}O_{6})}\cdot \;K_{1.1.1–1.1.2}$$

where $E_{GC(1.1.1/{{Nb}_{Li}^{I}O}_{6})}$ is the Coulomb interaction energy of a boron cation located in the ${Nb}_{Li}^{I}O_{6}$ face of a tetrahedron of Cluster 1.1.1, $E_{C(1.1.1/{Nb}_{Li}^{I}O_{6})}$ is a corresponding value from Figure 4, and *K_1.1.1–1.1.2_* is a coefficient calculated using the formula:

*K_1.1.1–1.1.2_* = *G*(Li_Li_) · *G*(Nb_Nb_) · *G*(Nb_Li_) · *G_1–4_*(V_V_) · *G*(V_Nb_) (8)

where *G*(Nb_Li_) is the population factor of Nb atoms occupying lithium octahedra, and *G*(V_Nb_) is the amount of vacant niobium octahedra calculated as:

*G*(V_Nb_) = 1 − *G*(Nb_Nb_) (9)

The coefficient *K_1.1.1–1.1.2_* is used because the structure of Clusters 1.1.1 and 1.1.2 is different only in the mutual location of defects along the Z axis; all other data (the amount of defects in the cluster, the site population factor for them) are the same. The coefficients are identical for Clusters 2.1.1 and 2.1.2 (*K_2.1.1–2.1.2_*), 3.1.1 and 3.1.2 (*K_3.1.1–3.1.2_*), 4.1.1 and 4.1.2 (*K_4.1.1–4.1.2_*), etc.

For crystals 5–8, Formula (8) has a slightly different way of *G*(V_v_) calculation (at an example of crystal 5):

*K_5.1.1–5.1.2_* = *G*(Li_Li_) · *G*(Nb_Nb_) · *G*(Nb_Li_) · *G_5–8_*(V_V_) · *G*(V_Nb_) (10)

For the remaining types of defects, the resulting Coulomb energy of the corresponding cluster was calculated according to the same algorithm as in Equations (7)–(10), but with the corresponding coefficients. We omitted them as their form is very similar and quite obvious. *G* values are taken from [39] or Table 3 and *E_C_* values from Figure 4, Figure 5, Figure 6 and Figure 7.

We obtained *E_GC(i)_* and summed them for crystals 1–4 in each position (crystal 1 is given as an example):

(11) $$E_{sumC(LN:Mg:B(1-HG)/{Li}^{I}O_{6})}=E_{GC(1.0.0/{Li}^{I}O_{6})}+E_{GC(1.1.1/{Nb}_{Li}^{I}O_{6})}+\;E_{GC(1.1.2/{Li}^{I}O_{6})}+E_{GC(1.2.1/{Li}^{I}O_{6})}+E_{GC(1.2.2/{Li}^{I}O_{6})}+E_{GC(1.3.1/{Mg}_{Li}^{I}O_{6})}+\;E_{GC(1.3.2/{Li}^{I}O_{6})}+E_{GC(1.4.1/{Li}^{I}O_{6})}+E_{GC(1.4.2/{Li}^{I}O_{6})}$$

Since in crystals 5–8, the defective structure is slightly different, the least members of Equation (11) are absent (crystal 5 is given as an example):

(12) $$E_{sumC(LN:Mg:B(5-SP)/{Li}^{I}O_{6})}=E_{GC(5.0.0/{Li}^{I}O_{6})}+E_{GC(5.1.1/{Nb}_{Li}^{I}O_{6})}+E_{GC(5.1.2/{Li}^{I}O_{6})}+\;E_{GC(5.2.1/{Li}^{I}O_{6})}+E_{GC(5.2.2/{Li}^{I}O_{6})}+E_{GC(5.3.1/{Mg}_{Li}^{I}O_{6})}+E_{GC(5.3.2/{Li}^{I}O_{6})}$$

**Figure 9:**
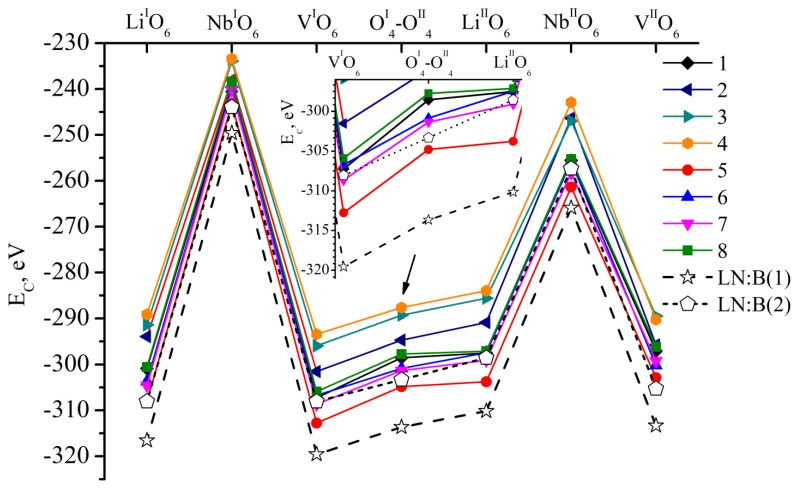
Dependence of *E_C_* on the position of the boron cation (seven positions), taking into account the population factors of sites in LN:Mg:B(1-4-HG); LN:Mg:B(5-8-SP); LN:B(1-HG); LN:B(2-SP) crystals [26]. A slanted arrow indicates which part of the graph is enlarged at an inset.

The fundamental calculation method is given in [26]. The calculation results are shown in Figure 9. For comparison, Figure 9 also shows the *E_C_* values for LN:B(1-HG) and LN:B(2-SP) crystals studied in [26].

Figure 9 shows that the total *E_C_* dependences of LN:Mg:B(1-4-HG) and LN:Mg:B(5-8-SP) crystals are 21–45 eV higher than *E_C_* of defect-free Clusters 1.0.0–8.0.0 (Figure 3). Such an increase in *E_C_* is due to all defects and their population coefficients.

The minimum value of *E_C_* for each individual dependence in Figure 9 is observed for the positions Li^I^O_6_, V^I^O_6_, O^I^_4_-O^II^_4_, Li^II^O_6_ and V^II^O_6_. This agrees well with our previously obtained results [26] and with the results in Figure 3. And the position of the dependences of *E_C_* on the localization of boron in different faces of the tetrahedrons has some features. For example, the minimum values of *E_C_* are characteristic of the LN:Mg:B(5-SP) crystal (Figure 9). The crystal has the minimum concentration of magnesium and boron of the two studied series of crystals, Table 1. Then, when the concentrations of magnesium and boron in the LN:Mg:B(6, 7, 8-SP) crystals increase, *E_C_* increases and takes on close values, Figure 9. If we move on to the LN:Mg:B(1-4-HG) crystals, *E_C_* behaves in the same way as in the case of the LN:Mg:B(5-8-SP) crystals. *E_C_* is minimal for the LN:Mg:B(1-HG) crystal with the minimal concentration of magnesium and boron in the series. The *E_C_* value of the LN:Mg:B(1-HG) crystal is approximately at the same level as the *E_C_* value of the LN:Mg:B(6, 7, 8-SP) crystals, Figure 9.

The minimum *E_C_* in each series of crystals is found in samples LN:Mg:B(1-HG) and LN:Mg:B(5-SP) (Figure 9) with the minimum concentration of magnesium and boron, Table 1. Two series were grown by dilution. That is, a nominally pure charge of congruent (R = 0.946) composition was added to the crucible after growing a crystal with the maximum concentration of magnesium and boron. This technique probably reduces the concentration of Nb_Li_, Nb_V_, Mg_Li_ and Mg_V_ defects in LN:Mg:B(4 → 1-HG) crystals and the Mg_Li_ defect in LN:Mg:B(8 → 5) crystals (Table 3 and work [39]). This is confirmed by the total occupancy of defect positions in crystals: 0.078 LN:Mg:B(4-HG) → 0.075 LN:Mg:B(3-HG) → 0.073 LN:Mg:B(2-HG) → 0.052 LN:Mg:B(1-HG); 0.053 LN:Mg:B(8-SP) → 0.065 LN:Mg:B(7-SP) → 0.060 LN:Mg:B(6-SP) → 0.051 LN:Mg:B(5-SP). If the decrease in magnesium concentration in the crystal series is explained by the dilution of the melt with a nominally pure charge, then for boron, this is also associated with its evaporation during the process of growing LN:Mg:B crystals.

In this case, the distribution coefficient close to 1 is favorable for the use of LN crystals. This is typical for LN:Mg:B(5-8-SP) crystals. For LN:Mg:B(1-4-HG) crystals, the distribution coefficient is significantly greater than 1, and it decreases rapidly over a fairly short concentration range [39]. Taking into account the data in Figure 9, we can say the following:

1. Growing LN:Mg:B crystals from a higher concentration to a lower one reduces the overall defectiveness of the crystals.
2. Despite the overall decrease in defectiveness, the LN:Mg:B(5-SP) crystal has the most stable structure. This is probably due to the absence of the Mg_V_ defect in it.

Co-doping with magnesium and boron promotes the evolution of the defect structure of LN:Mg:B crystals compared to single-doped LN:B. And the defectiveness of HG-doped crystals is higher. Indeed, Nb_Li_ and Nb_V_ defects are present in LN:B(1-HG) and LN:B(2-SP) crystals. The total population of these defects (0.025 and 0.028, respectively) is less than the total population of defects for LN:Mg:B(1-4-HG) and LN:Mg:B(5-8-SP) crystals (Table 3 and work [39]).

The minimum *E_C_* of 10 crystals LN:Mg:B(1-4-HG) and LN:Mg:B(5-8-SP), LN:B(1-HG) and LN:B(2-SP) is possessed by the crystal LN:B(1-HG), Figure 9. *E_C_* is slightly higher for the crystal LN:Mg:B(5-SP). *E_C_* of this crystal in almost all boron positions is lower than for the crystal LN:B(2-SP). The doping technologies for LN:B(2-SP) and LN:Mg:B(5-SP) crystals are the same, Figure 9. When monodoping is used, boron should be introduced into the LN crystal using a homogeneous method. And if the task is to obtain double-doped crystals (LN:Mg:B), then the technology of direct solid-phase doping is more advanced. In such crystals, the sequence of the main and doping cations along the growth axis will be preserved, and magnesium will not be introduced into vacant octahedra.

The contents of Mg and B in LN:Mg:B(3-HG) and LN:Mg:B(8-SP) crystals are close to each other. However, these crystals were obtained using different doping technologies. We compared the final calculation result of these crystals in Figure 9. The *E_C_* values for LN:Mg:B(8-SP) are much lower than those for LN:Mg:B(3-HG) for all boron positions. Moreover, the energy difference for each of the positions *ΔE_C_* = *E_C_*(LN:Mg:B(3-HG)) − *E_C_*(LN:Mg:B(8-SP)) is almost the same as the difference between the *E_C_* of LN:B(1-HG) and LN:B(2-SP). This finding confirms the importance of describing the complete doping and charge preparation technology and the growth parameters of doped LN single crystals when obtaining and interpreting any data, because crystals with the same dopant content obtained from charges of different genesis have different properties, from structure to distribution coefficient. Our conclusion confirms similar data for LN:Zn [63] obtained earlier. The conclusion about different properties of crystals with the same dopant concentration obtained by different technologies can partially explain the difference in the interpretation of many data concerning doped LN single crystals.

## 4. Conclusions

A study of two series of crystals LN:Mg:B(1-4-HG) and LN:Mg:B(5-8-SP) grown by the melt dilution method was carried out. Rietveld refinement for LN:Mg:B(2-HG) and LN:Mg:B(3-HG) was investigated for the first time. Models of boron cation localization in the faces of tetrahedrons of the LN structure were calculated. Analysis of the obtained models showed that the mechanism of magnesium incorporation into the crystal structure depends on the doping technology of the samples. It is shown that the HG method ensures the formation of a point defect Mg_V_ in the structure of LN:Mg:B(1-4-HG) crystals. It was not detected in LN:Mg:B(5-8-SP) crystals. All studied crystals were grown from a doped charge of congruent composition (R = 0.946) but have a stoichiometry value close to unity (0.965 < R < 1.014).

The presence of boron in the reaction mixture determines the type of realized vacancy model in the boron-containing crystal. This does not depend on the method of its introduction and the presence/absence of the second doping element. In such crystals, vacancies in the niobium position (V_Nb_^5−^) compensate for the excess positive charge of point structural defects.

In this work, based on the data of real crystals, 64 clusters of various configurations were constructed and calculated. It was experimentally established that the mutual arrangement of the main (Li, Nb) and impurity (Nb_Li_, Nb_V_, Mg_Li_, Mg_V_) structural units of the clusters depends on the doping technology, type, concentration of dopants and crystal growth method. The mutual arrangement of the main (Li, Nb) and doping (Nb_Li_, Nb_V_, Mg_Li_, Mg_V_) structural units of the clusters affects the localization of boron cations in the structure of LN:Mg:B crystals. In Clusters 1.1.1–8.3.2 and 1.4.1–4.4.2, it is most advantageous for the boron cation to be localized in the tetrahedron face common with V_Nb_. In this case, the absolute minimum of *E_C_* is achieved when boron is localized in the tetrahedron face common with V_Nb_^I^O_6_. In this case, point defects Nb_Li_, Mg_Li_ or Mg_V_ are located in the second layer of clusters. For Clusters 1.2.1–8.2.2, the opposite trend is observed: the absolute minimum of *E_C_* is recorded for the case when the Nb_V_ defect is localized in layer I, and the vacancy in the niobium position is localized in layer II. However, for Clusters 2.2.2 and 4.2.2, the trend characteristic of clusters with Nb_Li_, Mg_Li_ or Mg_V_ defects is preserved. And the localization of boron near V_Nb_ is due to the increase in the stoichiometry of boron-containing crystals (≈1), grown from a charge of congruent composition (0.946), with a charge difference (B^3+^ and V^I/II^_Nb_^5−^) and a steric factor.

Taking into account all the considered defect-containing and defect-free crystal clusters showed that the minimum *E_C_* in each series of grown crystals is possessed by the LN:Mg:B(1-HG) and LN:Mg:B(5-SP) samples, and the absolute minimum values are characteristic of the second crystal. This fact is very important for practice. This result is due to the fact that the melt was diluted when the crystals were grown, from the maximum concentration of dopants to the minimum. And this reduces the development of the defective structure of the crystals (reduction in the content of Nb_Li_, Nb_V_, Mg_Li_ and Mg_V_ in LN:Mg:B(4 → 1-HG) crystals; reduction in the content of the Mg_Li_ defect in LN:Mg:B(8 → 5-SP) crystals). The total population of defect positions in LN:Mg:B crystals decreases consistently (with the exception of the LN:Mg:B(8-SP) crystal). Thus, growing LN:Mg:B crystals from higher to lower concentrations reduces the total defectiveness of the crystals.

It has been established that double doping with magnesium and boron promotes the evolution of the defect structure of LN:Mg:B crystals compared to LN:B crystals. Its main contribution is reduced to a decrease in the concentration of Nb_Li_ defects.

Taking into account all the considered defect-containing and defect-free clusters of LN:Mg:B(1-4-HG) and LN:Mg:B(5-8-SP), LN:B(1-HG) and LN:B(2-SP) crystals helps to make an important conclusion. When monodoping is used, boron should be introduced into the LN crystal by the homogeneous doping method, but when there is a need to obtain co-doped crystals, the direct solid-phase doping technology is more advanced.

Studies have shown that LN:Mg:B crystals with a small number of structural defects can be obtained at relatively low dopant concentrations (~2.50 mol% MgO) by the SP method. The absence of Mg_V_-type defects in LN:Mg:B-SP crystals also supports this conclusion.

Our calculations also showed that LN:Mg:B(3-HG) and LN:Mg:B(8-SP) crystals with close dopant concentrations have different properties. Therefore, when comparing the properties of doped LN single crystals, it is critically important to describe all the details of the charge production, the technological parameters of growth for a complete understanding of the patterns of formation of these properties.

## Figures and Tables

**Table 1:** Concentration of magnesium and boron in LN:Mg:B(1-4-HG) and LN:Mg:B(5-8-SP) crystals [39].

Doping Technology	HG				SP			
Sample number	1	2	3	4	5	6	7	8
[Mg] in crystal, mol%	3.6	3.7	3.9	4.2	2.57	2.73	3.25	3.82
[B] in crystal cone, 10^−4^ wt%	0.59	1.0	1.7	9.0	0.42	0.76	1.13	2.07

**Table 2:** Possible designations of letters in the cluster number “A.B.C.”.

A	B	C
1	0 No defects 1 Nb_Li_ 2 Nb_V_ 3 Mg_Li_ 4 Mg_V_	0 No defects 1 Defect in layer I 2 Defect in layer II
2		
3		
4		
5		
6		
7		
8		

**Table 3:** The atomic coordinates (x/a, y/b, z/c), site occupancies (G, site occupancy), and *R*-factors obtained during Rietveld refinement for the samples LN:Mg:B(2, 3-HG) and LN:Mg:B(5, 6-SP).

	*G*	x/*a*	y/*b*	z/*c*		*G*	x/*a*	y/*b*	z/*c*
LN:Mg:B(2-HG): C(Mg) = 3.7 mol% *R_wp_*(%) = 6.76, *R_p_*(%) = 9.62					LN:Mg:B(3-HG): C(Mg) = 3.9 mol % *R_wp_*(%) = 6.59 *R_p_*(%) = 8.44				
Nb	0.956	0	0	0	Nb	0.925	0	0	0
O	1.0	0.0551	0.3249	0.0686	O	1.0	0.0547	0.3255	0.0634
Li	0.958	0	0	0.2950	Li	0.969	0	0	0.2783
Nb_Li_	0.014	0	0	0.2732	Nb_Li_	0.013	0	0	0.2670
Nb_V_	0.017	0	0	0.1550	Nb_V_	0.018	0	0	0.1109
Mg_Li_	0.017	0	0	0.2690	Mg_Li_	0.018	0	0	0.2900
Mg_V_	0.025	0	0	0.1212	Mg_V_	0.026	0	0	0.1470
LN:Mg:B(5-SP): C(Mg) = 2.57 mol%; *R_wp_*(%) = 6.78, *R_p_*(%) = 6.39					LN:Mg:B(6-SP): C(Mg) = 2.73 mol % *R_wp_*(%) = 8.12, *R_p_*(%) = 6.36				
Nb	0.957	0	0	0	Nb	0.955	0	0	0
O	1.0	0.0544	0.3473	0.0637	O	1.00	0.0642	0.3387	0.0643
Li	0.963	0	0	0.2807	Li	0.957	0	0	0.2821
Nb_Li_	0.015	0	0	0.2803	Nb_Li_	0.012	0	0	0.2900
Nb_V_	0.009	0	0	0.1100	Nb_v_	0.017	0	0	0.1100
Mg_Li_	0.027	0	0	0.2756	Mg_Li_	0.031	0	0	0.2877

## Data Availability

The raw data required to reproduce these findings are available from authors A.V.K., R.A.T. and D.V.M. on reasonable request due to privacy.
